# Comparative antioxidant efficacy of D-Cateslytin combined with modified triple antibiotic paste and calcium hydroxide: an *in vitro* study

**DOI:** 10.3389/fdmed.2026.1921120

**Published:** 2026-08-05

**Authors:** Alen Romel, Harish Shetty, Vidhyadhara Shetty, Shravan Kini, Nikhil Harikrishnan, Ashwini Prabhu

**Affiliations:** 1 Department of Conservative Dentistry and Endodontics, Yenepoya Dental College, Yenepoya (Deemed to be University), Mangalore, Karnataka, India; 2 Division of Cell and Molecular Biology, Central Research Laboratory, Srinivas University, Mangalore, India

**Keywords:** antioxidant activity, calcium hydroxide, D-Cateslytin, DPPH assay, intracanal medicaments, modified triple antibiotic paste

## Abstract

**Background:**

Successful endodontic treatment depends on effective elimination of microorganisms and the creation of a favorable biological environment for periapical healing. Conventional intracanal medicaments such as calcium hydroxide and Modified Triple Antibiotic Paste (MTAP) possess antimicrobial activity; however, their ability to modulate oxidative stress within the root canal environment is limited. D-Cateslytin, a peptide derived from Chromogranin A, has shown promising antimicrobial activity against various oral pathogens and may also contribute to antioxidant effects when used in combination with traditional intracanal medicaments.

**Aim:**

To evaluate and compare the antioxidant activity of D-Cateslytin in combination with calcium hydroxide and MTAP.

**Materials and methods:**

An *in vitro* experimental study was conducted using six experimental groups: control, D-Cateslytin, calcium hydroxide, MTAP, D-Cateslytin + calcium hydroxide, and D-Cateslytin + MTAP. The antioxidant activity was assessed by performing 2,2-diphenyl-1-picrylhydrazyl(DPPH) free radical scavenging activity and nitric oxide (NO) scavenging activity. The absorbance was measured at 517 nm for DPPH and 540 nm for NO.

**Statistical analysis:**

Data were analysed using one-way ANOVA followed by Bonferroni *post hoc* test (*p* < 0.05).

**Results:**

Both D-Cateslytin combination groups demonstrated greater antioxidant activity than calcium hydroxide and MTAP alone. Although the D-Cateslytin + MTAP group exhibited the highest antioxidant activity, it was not significantly different from D-Cateslytin alone or D-Cateslytin + calcium hydroxide.

**Conclusion:**

D-Cateslytin exhibited superior antioxidant activity compared to conventional intracanal medicaments. The addition of D-Cateslytin to calcium hydroxide and MTAP may enhance the biological properties of intracanal medicaments and could contribute to improved endodontic treatment outcomes.

## Key message

D-Cateslytin enhances the antioxidant activity of conventional intracanal medicaments, with D-Cateslytin-containing formulations demonstrated significantly greater antioxidant activity than conventional intracanal medicaments alone. The D-Cateslytin + MTAP formulation exhibited the highest numerical antioxidant activity, although it was not significantly different from the other D-Cateslytin-containing formulations. These findings suggest that peptide-based intracanal medicament combinations may represent a promising strategy to improve the biological environment and treatment outcomes in endodontic therapy.

## Introduction

The main aim of root canal therapy is to eliminate microorganisms within the root canal system and prevent reinfection to allow for periapical healing. However, there may be persistent microorganisms within the complex root canal anatomy despite thorough cleaning and shaping procedures. Contemporary endodontic practice does not allow leaving canals open between appointments. Thus, root canal medications are commonly used as an adjunct to mechanical instrumentation as well as for canal disinfection and controlling microbial growth during root canal therapy (1–3).

Calcium hydroxide, or Ca(OH)₂, has been considered the best medicament for use in the canal because of its high alkalinity, antimicrobial activity, and capacity to induce hard tissue formation at the periapical region (4). Nevertheless, its efficacy against resistant pathogens like *Enterococcus faecalis* and fungi is poor, thereby necessitating the use of other medicaments like Triple Antibiotic Paste (TAP) (5–7). In order to reduce the disadvantages of minocycline, like tooth discoloration and cytotoxicity, Modified Triple Antibiotic Paste (MTAP) was formulated by replacing minocycline with other antibiotics like clindamycin, which has shown better efficacy and biocompatibility compared to the conventional TAP (8–11).

In addition to the removal of microbes, the biological environment in the root canal system also has a significant impact on the success of the endodontic treatment. Oxidative stress, which occurs during the process of infection and inflammation, has been implicated in the damage to the tissues and the delayed healing in the periapical tissues. Recent experimental investigations have demonstrated the potential benefits of using antioxidant-modified irrigants and medicaments in the modulation of oxidative stress and the facilitation of periapical tissue healing (12–15). The antioxidant activity of the medicaments in the root canal system has been implicated in the enhancement of the bonding effectiveness of resin-based sealers to the dentin in the roots by neutralizing the oxidizing agents (5, 6, 14, 16).

Recent advances in biomedical research have emphasized the therapeutic potential of antimicrobial peptides as adjuncts in dental therapy. D-Cateslytin, which is derived from Chromogranin A, has been observed to possess potent antimicrobial activity against a broad spectrum of pathogens and stability in the biological environment (7, 9, 17). The effectiveness of D-Cateslytin in the presence of calcium hydroxide has been observed in several studies, which indicates the potential benefits of peptide-based adjuncts in intracanal therapy (8, 18).

Even though there have been several studies on the antimicrobial activity and cytocompatibility of various intracanal medications, there have been few studies on their antioxidant activity, especially in combination with antimicrobial peptides such as D-Cateslytin (5, 6, 11, 13, 17, 19). Thus, the aim of the present *in vitro* study was to evaluate and compare the antioxidant activity of D-Cateslytin in combination with calcium hydroxide and Modified Triple Antibiotic Paste. The present *in vitro* study consisted of six experimental groups, which were performed in triplicate. Group 1 constituted the control group, where DPPH + Methanol were used. Group 2 consisted of D-Cateslytin (78 µg/mL). The Group 3 samples were composed of Calcium Hydroxide (850 µg/mL). Group 4 samples were composed of Modified Triple Antibiotic Paste (100 µg/mL). Group 5 samples were composed of D-Cateslytin + Calcium Hydroxide. Group 6 samples were composed of D-Cateslytin + MTAP. Solutions were prepared in a stock concentration of 1 mg/mL and then diluted according to the experiment.

## Materials and methods

### Study design

The study was planned as an *in vitro* experimental study. The study was conducted at the Yenepoya Research Centre.

### Study groups

The study consisted of six experimental groups, each evaluated using three independent biological replicates. The groups were as follows: Group 1 was the control group, consisting of DPPH + methanol. Group 2 was D-Cateslytin 78 µg/mL. Group 3 was calcium hydroxide 850 µg/mL. Group 4 was Modified Triple Antibiotic Paste 100 µg/mL. Group 5 was D-Cateslytin + calcium hydroxide. Group 6 was D-Cateslytin + MTAP.

### Preparation of samples

The stock solutions of D-Cateslytin (GaloreTx Pharmaceuticals Private Limited, Bangalore, Karnataka, India), calcium hydroxide [Ca(OH)₂] (Prevest DenPro Limited, Bari Brahmana, Jammu & Kashmir, India), and Modified Triple Antibiotic Paste were prepared at a concentration of 1 mg/mL in distilled water. The combination groups were mixed immediately before performing the assays to ensure stability and consistency.

### Preparation of modified triple antibiotic paste (MTAP)

Modified Triple Antibiotic Paste (MTAP) was freshly prepared by combining ciprofloxacin (Cipla Ltd., Mumbai, Maharashtra, India), metronidazole (Abbott Healthcare Pvt. Ltd., Mumbai, Maharashtra, India), and clindamycin (Alkem Laboratories Ltd., Mumbai, Maharashtra, India) in equal proportions (1:1:1, w/w). The powdered antibiotics were thoroughly mixed and dissolved in sterile distilled water to obtain a stock solution of 1 mg/mL, from which the required working concentrations were prepared for the antioxidant assays.

### Nitric oxide scavenging assay

The nitric oxide scavenging activity of the test materials was determined *in vitro* using the Griess reagent method. The sodium nitroprusside solution was mixed with PBS (pH 7.4) and placed at room temperature for 150 min in the presence of varying concentrations of the test materials. Upon incubation, nitric oxide released from 10 mM sodium nitroprusside reacted with oxygen to yield nitrite ions. To the mixture, an equivalent volume of Griess reagent was added and incubated for 10 min in the dark. The mixture was then measured for absorbance at 540 nm using a microplate reader. The sodium nitrite solution was used to obtain a standard curve for measuring scavenging activity as a percentage compared to the control.

### DPPH free radical scavenging assay

The antioxidant activity of the test materials was determined using DPPH (2,2-diphenyl-1-picrylhydrazyl) free radicals. The DPPH solution (0.1 mM) prepared in analytical grade methanol were mixed with varying concentrations of the test materials and incubated for 30 min at room temperature in the dark. The mixture was then measured for absorbance at 517 nm. The percentage scavenging activity of DPPH free radicals was determined compared to the control.

### Statistical analysis

As this was an exploratory *in vitro* experimental study, a formal power-based sample size calculation was not performed. The statistical analysis plan was reviewed and approved by the study statistician. Data was coded in MS Excel and all statistical analysis was done using IBM SPSS 27 software. The significant difference between the groups was determined by one way ANOVA if the null hypothesis was rejected. If the null hypothesis was rejected, then the *post hoc* test was done for pair-wise comparison using the multiple comparison test. The significance level was set at *p* < 0.05.

## Results

The combination groups showed greater free radical scavenging activity compared to the individual medicament groups. The D-Cateslytin-containing groups demonstrated significantly greater free radical scavenging activity than calcium hydroxide and MTAP alone. Although the D-Cateslytin + MTAP group showed the highest mean antioxidant activity, it did not differ significantly from D-Cateslytin alone or D-Cateslytin + calcium hydroxide according to the Bonferroni *post hoc* analysis.

### DPPH free radical scavenging assay

**Figure 1:**
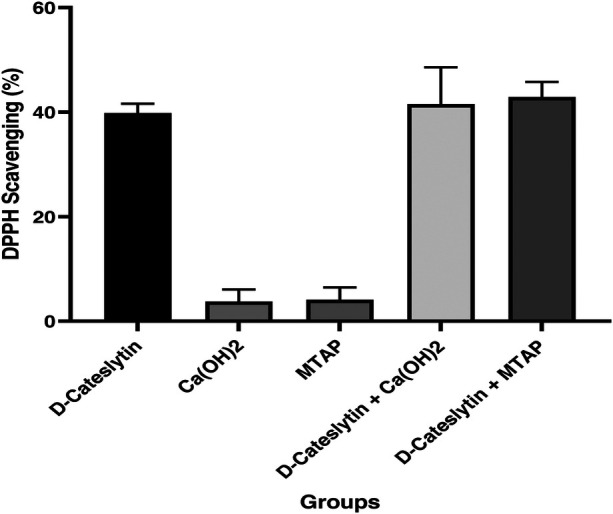
DPPH free radical scavenging activity (%). Values are represented as Mean ± SD (*n* = 3).

One-way ANOVA demonstrated a statistically significant difference in DPPH free radical scavenging activity among the experimental groups [F(4,10) = 87.72, *p* < 0.001] (Figure 1; Table 1). Bonferroni *post hoc* analysis showed that the D-Cateslytin, D-Cateslytin + calcium hydroxide, and D-Cateslytin + MTAP groups exhibited significantly greater antioxidant activity than the calcium hydroxide and MTAP groups, whereas no significant differences were observed among the D-Cateslytin-containing groups.

**Table 1:** DPPH free radical scavenging activity (%).

Group	Mean ± SD
D-Cateslytin	39.82 ± 1.80ᵃ
Ca(OH)₂	3.76 ± 2.31ᵇ
MTAP	4.10 ± 2.35ᵇ
D-Cateslytin + Ca(OH)₂	41.53 ± 7.05ᵃ
D-Cateslytin + MTAP	42.90 ± 2.91ᵃ

### Nitric oxide scavenging assay

**Figure 2:**
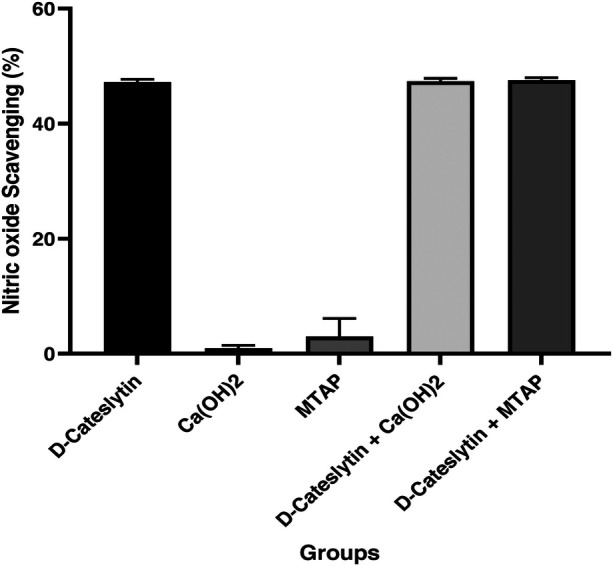
Nitric oxide scavenging activity (%). Values are represented as Mean ± SD (*n* = 3).

One-way ANOVA also revealed a statistically significant difference in nitric oxide scavenging activity among the experimental groups [F(4,10) = 854.76, *p* < 0.001] (Figure 2; Table 2). Bonferroni *post hoc* analysis demonstrated significantly higher nitric oxide scavenging activity in the D-Cateslytin-containing groups compared with calcium hydroxide and MTAP alone, while no statistically significant differences were observed among the D-Cateslytin-containing formulations.

**Table 2:** Nitric oxide scavenging activity (%).

Group	Mean ± SD
D-Cateslytin	47.22 ± 0.47ᵃ
Ca(OH)₂	0.94 ± 0.51ᵇ
MTAP	2.97 ± 3.15ᵇ
D-Cateslytin + Ca(OH)₂	47.36 ± 0.52ᵃ
D-Cateslytin + MTAP	47.56 ± 0.40ᵃ

Different superscript letters indicate statistically significant differences between the groups (*p* < 0.05).

Groups having the same superscript are not significantly different (One-way ANOVA using the Bonferroni *post-hoc* test).

## Discussion

Success in endodontic treatments involves not only the elimination of microbes but also the maintenance of a favorable biological environment within the root canal system. Persistent microbes and inflammatory mediators have shown potential in inducing oxidative stress, which might play a significant role in tissue destruction and slow periapical healing. Calcium hydroxide has traditionally been used as an intercanal medicament because of its antimicrobial properties and its potential in inducing hard tissue formation. Nevertheless, its poor efficiency against resilient microbes like *Enterococcus faecalis* has led to the development of alternative medicaments (4, 11).

Modified Triple Antibiotic Paste has been used as an alternative medicament for its enhanced antimicrobial properties and elimination of the disadvantages associated with conventional TAP. Previous studies have shown the potential of MTAP-based combinations in exhibiting high antibacterial properties and better biocompatibility compared to conventional TAP, in addition to minimizing cytotoxic effects on periapical-derived cells. Recent studies have shown the potential of MTAP-based combinations in reducing E. faecalis biofilm and exhibiting enhanced antimicrobial properties when used in combination with photodynamic therapy and bioceramic sealers (12, 18).

Antioxidants have been found to play a significant role in enhancing the interaction between dentin and resin-based sealers. This is due to the neutralization of oxidizing agents, thereby enhancing polymerization and sealer penetration into dentinal tubules (5, 6, 14). Intracanal irrigants and bioceramic-based medicaments containing high antioxidant properties have demonstrated enhanced antioxidant activity and retention of dentin microhardness compared to conventional products (14, 16, 19).

The current investigation aimed to determine the antioxidant activity of intracanal medicaments containing D-Cateslytin as an antimicrobial agent. Antimicrobial peptides such as D-Cateslytin have emerged as promising therapeutic agents for treating infectious diseases owing to their potent antimicrobial properties and resistance to degradation by infectious organisms (7, 9). Previous studies have demonstrated that D-Cateslytin containing calcium hydroxide exhibits potent antibacterial properties against endodontic pathogens such as Enterococcus faecalis (8, 18). This indicates that these peptides may play a significant role in enhancing the biological properties of conventional intracanal medicaments.

In the present study, the combination groups demonstrated higher antioxidant activity compared with the individual medicaments. This finding may be attributed to the enhanced effects between the antimicrobial peptide and the conventional medicaments. The D-Cateslytin-containing formulations exhibited comparable antioxidant activity, and all showed significantly greater antioxidant effects than calcium hydroxide and MTAP alone. Although the D-Cateslytin + MTAP group produced the highest numerical mean values, these were not significantly different from those of D-Cateslytin alone or D-Cateslytin + calcium hydroxide, suggesting that antibiotic-peptide combinations may offer additional biological advantages. These findings are consistent with reports showing that antioxidant-modified medicaments and peptide-incorporated calcium hydroxide can enhance biofilm eradication and preserve stem-cell function in regenerative models (13, 17, 19).

Recent evidence from regenerative endodontic research has further emphasized that the ideal intracanal medicament should achieve effective microbial elimination while preserving stem cell viability and promoting a favourable environment for tissue regeneration. Calcium hydroxide remains to be considered a valid intracanal medicament owing to its antibacterial properties and ability to form hard tissues; however, due to high alkaline nature, calcium hydroxide can be detrimental to stem cell viability at high concentrations. In the same way, modified triple antibiotic paste has proven to be highly effective in antimicrobial action, yet cytotoxicity and possible antibiotic resistance cannot be ignored. These new findings are consistent with the current study results where D-Cateslytin-containing preparations showed improved antioxidant activity (20, 21).

Although this study is useful to understand the antioxidant activity of intracanal medicaments, some aspects have to be kept in mind. Since this is an *in vitro* study, the results may not be comparable to the complex biological situations that occur clinically. Limitations of the study include the exclusive use of *in vitro* chemical antioxidant assays, small sample size, the absence of mechanistic molecular investigations and biological validation. More *in vivo* studies and clinical trials have to be done to assess the therapeutic potential of these combination products.

Within the confines of the *in vitro* study, the combination of D-Cateslytin with calcium hydroxide and Modified Triple Antibiotic Paste was seen to have increased antioxidant activity compared to the individual medicaments. Moreover, the highest antioxidant activity was seen in the D-Cateslytin + MTAP group, followed by the D-Cateslytin + calcium hydroxide group. This shows that the combination formulations exhibited enhanced antioxidant activity than the conventional medicaments alone. This study shows that the addition of D-Cateslytin to calcium hydroxide or MTAP could potentially improve the biological activity of conventional intracanal medicaments by reducing oxidative stress, thereby enhancing the healing of periapical tissues and sealer/dentin interactions in endodontic therapy.

## Conclusion

Within the limitations of this *in vitro* study, the combination formulations demonstrated greater antioxidant activity than the individual intracanal medicaments, with D-Cateslytin combined with Modified Triple Antibiotic Paste showing the highest free radical scavenging activity. These findings suggest that incorporating D-Cateslytin into conventional intracanal medicaments may be a promising strategy for enhancing their antioxidant properties. However, further studies evaluating antimicrobial activity, cytocompatibility, and clinical performance are required before any conclusions regarding their biological effectiveness or impact on endodontic treatment outcomes.

## Data Availability

The raw data supporting the conclusions of this article will be made available by the authors, without undue reservation.
